# Timely Colonoscopy After Positive Fecal Immunochemical Tests in the Veterans Health Administration: A Qualitative Assessment of Current Practice and Perceived Barriers

**DOI:** 10.14309/ctg.0000000000000438

**Published:** 2022-02-19

**Authors:** Ashley C. Mog, Peter S. Liang, Lucas M. Donovan, George G. Sayre, Aasma Shaukat, Folasade P. May, Thomas J. Glorioso, Michelle A. Jorgenson, Gordon Blake Wood, Candice Mueller, Jason A. Dominitz

**Affiliations:** 1 Veteran Affairs Puget Sound Healthcare System, Seattle, Washington, USA;; 2 University of Washington, Seattle, Washington, USA;; 3 Veteran Affairs New York Harbor Healthcare System, New York, New York, USA;; 4 NYU Langone Health, New York, New York, USA;; 5 Minneapolis Veteran Affairs Healthcare System, Minneapolis, Minnesota, USA;; 6 University of Minnesota, Minneapolis, Minnesota, USA;; 7 Veteran Affairs Greater Los Angeles Healthcare System, Los Angeles, California, USA;; 8 University of California, Los Angeles, California, USA;; 9 CART Program, Office of Quality and Patient Safety, Veterans Health Administration, Washington, DC, USA;; 10 Bay Pines Veteran Affairs Healthcare System, Bay Pines, Florida, USA.

## Abstract

**INTRODUCTION:**

The Veterans Health Administration introduced a clinical reminder system in 2018 to help address process gaps in colorectal cancer screening, including the diagnostic evaluation of positive fecal immunochemical test (FIT) results. We conducted a qualitative study to explore the differences between facilities who performed in the top vs bottom decile for follow-up colonoscopy.

**METHODS:**

Seventeen semistructured interviews with gastroenterology (GI) providers and staff were conducted at 9 high-performing and 8 low-performing sites.

**RESULTS:**

We identified 2 domains, current practices and perceived barriers, and most findings were described by both high- and low-performing sites. Findings exclusive to 1 group mainly pertained to current practices, especially arranging colonoscopy for FIT-positive patients. We observed only 1 difference in the perceived barriers domain, which pertained to primary care providers.

**DISCUSSION:**

These results suggest that what primarily distinguishes high- and low-performing sites is not a difference in barriers but rather in the GI clinical care process. Developing and disseminating patient education materials about the importance of diagnostic colonoscopy, eliminating in-person precolonoscopy visits when clinically appropriate, and involving GI in missed colonoscopy appointments and outside referrals should all be considered to increase follow-up colonoscopy rates. Our study illustrates the challenges of performing a timely colonoscopy after a positive FIT result and provides insights on improving the clinical care process for patients who are at substantially increased risk for colorectal cancer.

## INTRODUCTION

Screening is effective in reducing mortality from colorectal cancer (CRC) (1), the second leading cause of cancer death in the United States (2). Annual screening with the noninvasive fecal immunochemical test (FIT) is one of several recommended screening strategies (3). However, previous studies have found that the proportion of individuals with a positive (abnormal) FIT who undergo diagnostic colonoscopy to assess for the presence of cancer is variable, ranging from 30% to 82% (4). The gap in evaluation is especially concerning as approximately 4% of individuals with a positive FIT are found to have CRC at the time of colonoscopy (5). Therefore, current guidance recommends that screening programs should aim for at least 80% of individuals with an abnormal FIT undergo timely diagnostic colonoscopy (5). Furthermore, delays in colonoscopy after abnormal FIT beyond 6–12 months have been associated with significantly increased CRC mortality (6,7).

In 2017, the Veterans Health Administration (VA) introduced the Colorectal Cancer Screening/Surveillance (CRC S/S) reminder system to help address gaps in the process of CRC screening and surveillance, including diagnostic evaluation of a positive FIT. To assess the effect of the new reminder system on time to colonoscopy after a positive FIT, a working group formed by the VA National Gastroenterology Program measured the proportion of positive FIT results between January 2017 and September 2018, which were followed by a colonoscopy within 6 months at each VA facility (130 in total). The working group observed substantial variation on the 6-month colonoscopy completion, with the top decile of facilities having a mean 6-month colonoscopy Kaplan-Meier completion of 59%, compared with 31% in the bottom decile. To explore these differences and identify best practices, we conducted a qualitative study of high- and low-performing sites, sampled from the top and bottom decile facilities.

## METHODS

This was an operational quality improvement project commissioned and sponsored by the VA National Gastroenterology Program and was exempted from institutional review board review. We purposively sampled sites with high vs low rates of colonoscopy completion after positive FIT. After reviewing colonoscopy rates at all 130 VA sites nationally adjusted for patient demographics (age, sex, race, ethnicity, rurality, distance to VA, and insurance status), comorbidities (myocardial infarction, congestive heart failure, peripheral vascular disease, cerebrovascular disease, dementia, chronic obstructive pulmonary disease, connective tissue disease, peptic ulcer disease, mild/severe liver disease, diabetes, paraplegia/hemiplegia, renal disease, cancer, and metastatic carcinoma), prior visit history, and facility characteristics (complexity, gastroenterology patient volume, and colonoscopy volume), we identified 14 high performers and 14 low performers. We contacted the gastroenterology (GI) section chiefs at each site to request interviews with individuals who were knowledgeable about the positive FIT follow-up process. A total of 17 semistructured telephone interviews were completed from February through December 2019 at 9 high-performing (57% median unadjusted Kaplan-Meier rate for the 6-month colonoscopy) and 8 low-performing sites (34% median unadjusted rate) with individuals in several different roles (Table 1). We followed conventional practice regarding sample size in qualitative research and conducted interviews until we reached saturation in main content areas (8,9). Interview guides (see Supplementary Digital Content 1, http://links.lww.com/CTG/A753) were designed to elicit rich descriptions and perspectives regarding the process of performing colonoscopies for Veterans after a positive FIT at each site, using questions sequenced from least constrained to most constrained and follow-up probes grounded in participant's verbatim language (10).

**Table 1.** Interviewee roles by site

Interviewee role	Low-performing site	High-performing site
GI section chiefs	5	5
GI coordinators	1	3
Other GI staff	2 gastroenterologists	1 nurse

GI, gastrointestinal.

Two interviewers (C.M. and L.M.D.) with 2–4 years of experience in qualitative interviews were blinded to site performance status (high or low). All interviews were audio-recorded and transcribed by staff who were also blinded to site performance status.

Initial data analysis and collection were performed concurrently, and analysts were also blinded to site performance status. Using the Matrix Analysis (11), an approach well-suited to health system research (12,13), we organized the data into *a priori* domains, including current practices, perceived barriers and facilitators, community care (non-VA care), and patient experience. Data analysts reviewed interview transcripts and placed salient results into domain categories. Under each domain, categories and subcategories were identified from the data and applied to quotes in each domain section of the matrix (14). After consensus on categorizations was reached, site performance status was unblinded. We considered results as distinct to high- or low-performing sites if there were 2 or more sites with the same performance status with a particular result and no site in the other performance status group reporting that perceived barrier or facilitator.

## RESULTS

We identified findings in 2 domains: current practices and perceived barriers. Most findings were described across both high- and low-performing sites, although there were findings exclusive to high- or low-performing sites, described further and in Table 2. Note that an absence in one category is not necessarily indicative of an absence of that practice at a site but rather indicates inclusion at the sites noted.

**Table 2.** Domains, categories, and illustrative quotes regarding the management of FIT-positive patients

Domain: category	Subcategory	Illustrative quotes
Current practices: managing FIT results	Primary care follows up with patients who miss colonoscopy (exclusive to low sites)	We get a lot of no shows, cancellations… it was supposed to be that Primary Care is supposed to explain the different reasons [for colonoscopy after FIT+] to the patient, but they don't. They explain nothing to the patient, because they don't have time.—GI chief, low site
	Multiple ways to notify providers about results	There's many ways they [PCPs] get notified, first it's visible on the patient's cover sheet, where they open the patient's sheet and look at the list of reminders, it would be there.—GI chief, high site
	Primary care management of FIT results	…the results go back to the ordering provider ….But there are still some providers who still get that [FIT] test for other reasons outside of screenings, and I think that often has influenced why a patient is or isn't referred to our section, and why a patient wants to undergo a colonoscopy or doesn't want to undergo a colonoscopy.—GI chief, low site
	Education of primary care	The educational piece about doing the test in the first place is there's dialogue… they go through an educational process about the role of FIT testing, and what you do if it's positive.—GI chief, high site
Current practices: arranging colonoscopy for FIT-positive patients	Avoiding community care for FIT-positive patients (exclusive to low sites)	We typically don't [send to community care]. If it's a FIT positive, we take care of it here… if it's a FIT-positive consult or a FIT-positive indication, we typically will make arrangements to have them seen within 30 d.—GI chief, low site
	E-consults from primary care to GI (exclusive to high sites)	Well, we've recently implemented the E-consulting, so if they meet certain criteria, we don't have to necessarily have them come in for 2 different visits; 1 for procedure and 1 for clinic. So that has helped speed things along.—GI coordinator, high site
	GI knowledge in the Community Care (non-VA care) Office (exclusive to high sites)	…what we found was, having a few champions, and interestingly, a few people from the GI Section went over to start working in the Community Care Office, so they already knew quite a bit about FIT positive and colonoscopy… so they were a lot more knowledgeable and a lot more effective in getting these scheduled.—GI chief, high site
	GI tracks community care consults (exclusive to high sites)	If the Veteran elects to go to community care, we still will have reviewed that request and approved it to go to community care… So, everything, at least in [VA Facility], that goes out to the community, we touch it as it goes out, and then we touch it as it comes back in so that we can reset the Clinical Reminder.—Section chief, high site
	General patient education about CRC (exclusive to high sites)	… we want to develop a series of web-based tools as well as things the Veterans can look at…kiosks…so that each month, like March is colorectal cancer month, the Veterans who are attending here will get some additional emphasis on the importance of colorectal cancer screening, obviously in March, but it's all year round.—Section chief, high site
	Patient education about colonoscopy before procedure	We're doing education phone calls about a week before… That's improved our show rate a lot… there was a week or 2 where we were so short nurses in September… so we didn't have our education team making the phone calls, so our no-show and late cancel went way up.—GI chief, high site
	GI coordinator ensures that all studies are completed	I'm notified that the patient either cancelled or no-showed, I try and contact them by phone, and if successful, I get them rescheduled.—GI coordinator, high site
	Tracking FIT results using gap reminder	… we update the gap reminder, and we cc the Primary Care person. So everybody knows what's done, and we write in the comment note, we have what the procedure was or why we did it, and then when the biopsy comes back, we add to that Endo note, what the biopsy was and what our recommendation is.—GI chief, low site
Perceived barriers: to colonoscopy	Coordination issues between GI and primary care	Although I sound like I'm mad at Primary Care, I am, but it's not their fault. First of all, they have a lot of turnover… Secondly, in 15 min, they don't have time to do all of the things that Central Office wants them to do. I understand that, they can't.—GI chief, high site
	Underscheduled endoscopy unit	The biggest obstacle that I have noticed since I have taken over as Chief of this VA is that we just lack the dedication; dedicated schedulers. They're so pulled in so many directions… they're just not able to get patients in the way I would like them to.—GI chief, high site
	Lack of needed resources	…no matter what you're going to do, as good as it sounds, it's going to need either space, it's going to need resources… or it's going to require RNs, NPs, or providers… We're all stressed, we all can't deal with it, because they didn't give us the tools to be successful with the gap reminder.—GI chief, low site
Perceived barriers: for PCPs	Bandwidth: Number of mandatory clinical reminders (exclusive to low sites)	…when we get a GI consult, I'm telling you, that patient has 4 other consults. And then, I wrote to [the PCP], I said “don't send a GI consult for a screening colonoscopy at the same time you're sending a Pulmonary consult for shortness of breath and a Cardiac consult for chest pain,” you know? I'm going to deny the consult. I'm not doing a person with chest pain or shortness of breath for a screening colonoscopy. I sound frustrated, I am frustrated, because the system is a broken system.—GI chief, low site
	Bandwidth: shared decision-making	It's rare that a Primary Care Doctor has enough time during a routine Primary Care encounter to cover all of these issues. So often times these are left as secondary conversations, or no conversation, and the testing is done really without a lot of education involved.—Section chief, high site
	Knowledge	In the initial counseling, it's pointless to send a FIT test if you don't understand that if it's positive, that the consequence of a positive test is a colonoscopy.—GI coordinator, low site
Perceived barriers: for patients	Concerns about safety, invasiveness, or fear of procedure	… there's the occasional patient that I see…where it's very clear that the patient refused to go further, even if the test was positive, they refused to have colonoscopy. I discontinued a Veteran who has now no-showed a number of times, and he's 70, and I sent a letter back to his Primary Care and said, “look, we're going to have to close this consult, you're going to have to talk to him again and ask him if he really wants to do that the next time you see him.”—GI chief, high site
	Inability to discuss with provider	…the patient either doesn't understand the importance, doesn't want to undergo colonoscopy, said it was never discussed and that they want to wait until they see their doctor again in 6 mo to discuss it. So, the ball gets dropped at the patient level quite often.—GI chief, high site
	Health literacy	You know, we try to do education with the patients. Sometimes they don't really understand why they need to have this done. So, just giving them the information so that they can make an informed decision. That's still what they choose, but a lot of times they don't have all of the information that they need to really make a good decision.—Nurse, high site
	Social support	Most of the time, I'd say greater than 50% of the time, patients are interested to participate in their healthcare. It's really the social barriers for them, in terms of getting somebody to drive them and just the logistics of doing the test that are challenging… it can take 2 or 3 times and lots of rescheduling to actually get them in to get it completed.—Section chief, high site

CRC, colorectal cancer; FIT, fecal immunochemical tests; GI, gastrointestinal; PCP, primary care physician; VA, Veterans Health Administration.

### Current practices

#### Managing results

FIT results are managed in a variety of ways across the interviewed sites. Six facilities reported communicating results only to the ordering provider, typically the primary care provider (PCP), whereas others also report abnormal results to GI (n = 4). Two sites reported multiple electronic health records reminders are used to alert providers to abnormal FIT results. Six facilities identified an individual responsible for tracking FIT-positive patients and helping to coordinate care for those patients.

Management of FIT results in primary care included involvement of the patient aligned care teams, a patient-centered medical home model, to manage their teams' results (e.g., during daily huddles; n = 3). Education of primary care was identified as another salient component (n = 5), which included education about the importance of following up abnormal FIT results, dissemination of the appropriate clinical workflow within patient aligned care teams, and GI direction of early management of FIT results.

Only low-performing sites (n = 2) reported that it is the responsibility of primary care to manage patients with positive FIT who missed their colonoscopy appointment. Those sites also reported that primary care may not address the reason for no-shows or give patients the information they need to understand the importance of GI evaluation for the abnormal FIT result.

#### Arranging colonoscopy for FIT-positive patients

Various strategies were used to support colonoscopy access, which included management plans for patients who failed to show for their preprocedure and colonoscopy appointments. One low-performing site arranged hotel accommodations for patients without transportation. One high-performing and 1 low-performing site also used flexible appointment times (e.g., offering alternate times, such as Saturday colonoscopy appointments, or allowing follow-up appointments to be scheduled up to a year in advance) for Veterans who were traveling longer distances.

Direct access colonoscopy, in which patients who meet certain medical criteria can forgo a preprocedure visit, was used by both low- and high-performing sites. A GI coordinator at a high-performing site discussed with Veterans what will happen in the case of a positive FIT result before the test was ordered. Seven sites described use of GI education for all patients after colonoscopy referral, which usually consisted of a phone call from either a GI nurse or provider before the procedure.

Other GI-specific current practices included having a coordinator ensure all studies were completed and prioritizing FIT-positive patients for colonoscopy (n = 5). Two sites reported using the recently introduced CRC S/S website, which provides an updated report of patients with positive FIT results who have not received a colonoscopy in each facility, to identify patients for targeted outreach.

Our analysis identified 4 facilitators exclusive to high-performing sites. Two sites used e-consulting from primary care to GI, which eliminated a preprocedure clinic visit for patients who met certain criteria. Three sites noted that the presence of specific GI knowledge about the importance of this issue in the Community Care Office (which manages referral to non-VA providers) helped to schedule colonoscopies in a timely manner. Five sites referenced have VA GI staff track all Community Care consults both preprocedure and postprocedure to keep VA providers aware of their patients' colonoscopy results from outside providers. Four sites also reported working on solutions for patient education and support, including educational exhibits (n = 2), online educational videos (n = 1), and patient navigators (n = 1).

The only practice exclusive to low-performing sites (n = 2) was avoidance of community care referral for colonoscopy after a positive FIT. The reasons cited for this practice included difficulty in retrieving external procedure reports and the ability to perform endoscopy in the VA within 30 days in most cases.

### Perceived barriers

#### GI perceived barriers to colonoscopy

Several perceived barriers for colonoscopy after positive FIT were reported. Challenges within primary care accounted for some of the barriers. A GI at a low-performing site described a sincere lack of coordination between GI and primary care, while a GI chief at a high-performing site described high physician turnover in primary care. Other sites mentioned issues handling CRC S/S reminders (n = 3), including that it is a time-intensive process, patients who refuse a procedure are not removed from the tracking list, and patients are receiving reminders for FIT despite undergoing colonoscopy the previous year (which may occur if a colonoscopy was performed outside of the VA and was not documented). Having a chronically underscheduled endoscopy unit was cited as a barrier to care, partially attributed to the lack of committed schedulers. Four sites described a lack of resources to perform colonoscopy for FIT-positive patients, including space and staff. Four other sites mentioned a lack of pre-FIT patient counseling and FIT being ordered on patients who would never agree to a colonoscopy.

### Perceived PCP barriers

Two types of barriers for PCPs were identified: challenges with bandwidth and knowledge. Sites reported a perception of bandwidth barriers for PCPs, specifically the number of mandatory clinical reminders (exclusive to low performing sites; n = 2), meaning that PCPs are often juggling many responsibilities (alerts and/or consultations) with insufficient time. Sites also shared the perception that because PCPs are overtaxed, they are often unable to educate patients and engage in shared decision-making about the appropriate follow-up for an abnormal FIT result (n = 5).

Reported PCP knowledge barriers included inappropriate use of FIT, such as repeating an FIT after a positive result. A GI chief from a high-performing site described how unrealistic clinically indicated dates (date deemed clinically appropriate by a referring provider, which is used to calculate appointment wait times) pushed colonoscopies to community care, when they could be performed within the VA if the referring PCP understood the workflow of the GI service. Finally, 2 respondents noted that a positive FIT always requires a colonoscopy, and PCPs do not always seem to understand or facilitate this standard when they receive the alert.

### Perceived patient barriers

Sites reported perceived patient barriers that applied to both high- and low-performing sites. Two respondents stated that some patients do not seem motivated to complete the procedure, while 7 sites reported that patients had concerns about the safety or invasiveness of the procedure. A few sites mentioned patient embarrassment (n = 1) or the inability to discuss the need for colonoscopy with the provider (n = 3). Four sites reported low health literacy as a barrier to care, such as when patients may not understand the need for a colonoscopy. Lack of social support (n = 6) and reliable transportation (n = 4) were cited as additional patient barriers.

## DISCUSSION

In this qualitative study involving GI physicians and staff at 17 facilities across the VA healthcare system, we examined the process of managing patients with positive FIT results at both high- and low-performing sites. While many findings were common to both groups, we identified some that were exclusive to either high- or low-performing sites. Only high-performing sites discussed using e-consults to reduce the need for a preprocedural clinic visit for select patients, having GI knowledge in the office that facilitates Community Care referrals outside of the VA, having GI track all Community Care consults, and developing resources for patient education and support. By contrast, only low-performing sites described having solely primary care follow-up patients who miss colonoscopy appointments, avoiding Community Care referrals, and seeing the large number of mandatory clinical reminders as a barrier for PCPs.

Most of the findings that were exclusive to 1 group were within the current practices domain and the category of arranging colonoscopy for FIT-positive patients. We observed only 1 difference in the perceived barriers domain, which pertained to PCPs. These results suggest that what primarily distinguishes high- and low-performing sites is not a difference in the perception of barriers but rather a difference in the GI clinical care process. In particular, developing and disseminating patient education materials about the importance of diagnostic colonoscopy, eliminating an in-person precolonoscopy visit when clinically appropriate, and involving GI in missed colonoscopy appointments and outside referrals are practices that every facility should consider.

Privett and Guerrier (15) have estimated that PCPs would require an infeasible 8.6 hours per working day just to deliver preventive services recommended by the US Preventive Services Task Force; CRC screening was the most time-consuming of all recommendations at 34 minutes. Thus, systems-level interventions to improve the process of obtaining a colonoscopy after a positive FIT result will likely offer the most effective solution. For instance, organized screening has a number of advantages compared with an opportunistic screening (16), and a recent international survey of 35 screening programs found much higher follow-up colonoscopy rates in organized programs than in opportunistic ones (17). In addition, both the survey and a systematic review found that patient navigation and provider-level reminders are effective for increasing colonoscopy completion after a positive FIT result (17,18). In the survey, those programs that used patient navigation and PCP reminders had an 11% and 12% increase in colonoscopy completion at 6 months, respectively, compared with those that did not.

Given that approximately 4% of all individuals with a positive FIT are found to have CRC and that delays in colonoscopy are associated with adverse outcomes (5–7), it is important for healthcare systems to address barriers to timely colonoscopy. In 2005, the VA conducted a yearlong quality improvement initiative at 21 facilities that decreased the average time to follow-up colonoscopy from 129 to 103 days (19). Data from a 2007 VA-wide survey suggested that developing quality improvement infrastructure and improving care delivery processes seemed to be effective strategies for increasing follow-up colonoscopy rates (20). Subsequently, Partin et al. showed that predictors of colonoscopy completion after an abnormal fecal occult blood test (FOBT) result included both organizational factors (e.g., notifying GI directly about abnormal results, using written and verbal colonoscopy preparation instructions, and opt-in scheduling (21,22)) and patient factors such as age and/or comorbidity (23).

Despite ongoing research and operational efforts within the VA, we found that facility colonoscopy completion rates within 6 months at the highest performing facilities averaged only 59%. In a recent study evaluating wait times for colonoscopy after a positive FOBT result in the VA, Adams et al. found that wait times have been stable between 2008 (median 50 days) and 2015 (median 52 days) (24). Since 2015, the VA has expanded access to community care, including requirements for offering non-VA care whenever the VA cannot provide an appointment within 28 days of the clinically indicated date. Therefore, the large proportion of Veterans who do not receive colonoscopy within 6 months of a positive FIT result cannot simply be explained on the basis of lack of availability of colonoscopy appointments.

Social barriers, including the absence of a companion or reliable transportation after procedural sedation, were cited by both high- and low-performing sites. Similar problems in other institutions and potential solutions have been described (25,26). The VA developed an internal postsedation care and discharge toolkit in 2019 specifically to address these issues, although the potential effect of the toolkit would not be reflected in our findings because it occurred after the study period.

A lack of follow-up colonoscopy is also a common problem outside of the VA, and rates of 50% or less have been reported in other US institutions and European nations (27,28). A previous qualitative study of 30 patients with an abnormal FOBT and 30 PCPs explored reasons for a lack of follow-up colonoscopy in Ontario, Canada (29). Two of the 4 reasons highlighted by the Canadian study—patients' fear of colonoscopy and breakdown in communication of abnormal FOBT results or colonoscopy appointments—were also identified in our study. Another qualitative study involved 17 patients who did not undergo colonoscopy after a positive FIT result in the Dutch national screening program, which notifies individuals of their positive FIT result along with a premade appointment for colonoscopy consultation within 2 weeks (30). The Dutch program had a follow-up colonoscopy rate of greater than 85%. A common theme was that participants preferred a more personalized approach, including discussion of options besides colonoscopy, rather than the highly streamlined referral process. However, it is almost certain that the efficient referral process is a major reason behind the program's impressive follow-up colonoscopy rate. It is unclear whether a similar approach would be effective in the VA because opt-out scheduling has been previously associated with a greater number of missed and canceled colonoscopy appointments (22). Finally, a qualitative study of 21 PCPs and staff members at a safety net hospital in Seattle identified social determinants of health (e.g., transportation availability), organizational factors (e.g., care coordination), and patient cognitive factors (e.g., bowel preparation challenges) as barriers to colonoscopy completion (31). All these themes were also captured in our study, which complement and extend the existing literature that has focused on PCPs and patients.

Kaiser Permanente Northern California, another large integrated healthcare system, increased their 6-month follow-up colonoscopy rate from 67% in 2006–2008 to 83% in 2013–2016 (32). They implemented a number of interventions, including expanding endoscopic capacity, setting a goal of ≥80% of FIT-positive patients completing colonoscopy within 30 days (with some exclusions), imposing financial penalties for sites that fail to meet goals, assigning responsibility of managing FIT-positive patients to GI, and standardized patient navigation. Although it is unclear which of these interventions had the greatest effect, setting an explicit system-wide goal seems to be necessary step for the VA and any system that strives to improve follow-up colonoscopy rates.

A few limitations of our study should be noted. First, as with all qualitative data, one should not assume there is a causal relationship between the factors exclusive to a group and the performance status of that group. Second, we did not collect demographic information on participants or the patients they care for and, therefore, cannot provide any insights related to these characteristics. Third, certain aspects of this study reflect processes specific to the VA and may not be applicable to other healthcare systems. Fourth, we did not interview PCPs or patients to gain their perspectives of this process. Finally, the facilities that responded to interview requests may not represent the other low- or high-performing facilities.

In conclusion, timely diagnostic colonoscopy after abnormal FIT is a key component of any CRC screening program, although the proportion of Veterans completing this examination falls short of benchmarks. Our qualitative study on the challenges of performing a timely colonoscopy after a positive FIT result provides important insights into how to improve the clinical care process for these patients who are at significantly increased risk for CRC and CRC mortality.

## CONFLICTS OF INTEREST

**Guarantor of the article:** Jason A. Dominitz, MD, MHS.

**Specific author contributions:** A.C.M.: planning and conduct of study, interpretation of data, and drafting and critical revision of the manuscript. P.S.L.: planning of study, interpretation of data, and drafting and critical revision of the manuscript. L.M.D., G.G.S., C.M., and J.A.D.: planning and conduct of study, collection and interpretation of data, and critical revision of the manuscript. A.S., F.P.M., M.A.J., and G.B.W.: planning of study and critical revision of the manuscript. T.J.G.: collection and interpretation of data and critical revision of the manuscript. All authors approved the final draft.

**Financial support:** The Veterans Health Administration National Gastroenterology Program office commissioned and sponsored this evaluation.

**Potential competing interests:** None to report.

**Disclaimer:** This study is the result of work supported in part by resources from the Veterans Health Administration. The content is solely the responsibility of the author and does not represent the views of the US Department of Veterans Affairs or the US government.

**Study Highlights**

**WHAT IS KNOWN**

✓ Individuals with a positive fecal immunochemical test (FIT) have a substantially increased risk of colorectal cancer.
✓ Follow-up colonoscopy rates after positive FIT results are often suboptimal, but differences between low- and high-performing facilities are unclear.

**WHAT IS NEW HERE**

✓ The main differences between low- and high-performing facilities relate to clinical processes, such as managing FIT results and arranging colonoscopies.
✓ Improving patient education, eliminating precolonoscopy visits when clinically appropriate, and involving gastroenterology service in navigating missed appointments may increase follow-up rates.

## Supplementary Material

SUPPLEMENTARY MATERIAL
