# Supplementary material for: Timely Colonoscopy After Positive Fecal Immunochemical Tests in the Veterans Health Administration: A Qualitative Assessment of Current Practice and Perceived Barriers
Source: Clin Transl Gastroenterol. 2022 Feb 19;13(2):e00438. doi: 10.14309/ctg.0000000000000438 (PMC8865517; doi:10.14309/ctg.0000000000000438)
Supplement: SUPPLEMENTARY MATERIAL [file ct9-13-e00438-s001.pdf]

Providing Colonoscopies for Veterans Who are FIT Positive
Interview Guide

Interviewer Name:

Date:

Time Start:
Time End:

Hello [Mr./Ms. interview participant name],

My name is [interviewer name].

We are talking to VA providers, administrators and staff to better understand how sites are providing colonoscopies to Veterans who are FIT OR FOBT (fecal occult blood test) positive.

We won't identify you as a participant, nor will we identify your site in any of our reports.

The call will take approximately 30 minutes.

Your participation in this interview is voluntary and confidential and your anonymity will be protected. You can stop the interview at any time, and may let me know if you'd rather not answer a particular question.

Do you have any questions?

In order to make sure we capture all of the information you give us, we would like to record this call. The audio-file for the recording will be stored directly to restricted access file on the VA intranet. Is this okay with you? **[Hit record button.]** Okay, to confirm, I'm starting the recording. Is this ok with you?

Grounded prompts: If responses are limited or require clarification, probes may be used to elicit more detailed responses. Probes should use words or phrases presented by the participant using one of the following formats:
1. What do you mean by ____________?
2. Tell me more about ____________?
3. Give me an example of ____________?
4. Tell me about a time when ____________?
5. Who ____________?
6. When ____________?
7. What happens/ed next/after ____________?
8. What happens/ed before ____________?
9. Can you walk me through ___________?

1. What is your role concerning colorectal cancer screening (e.g. Primary Care Provider, Gastroenterologist, Quality Manager, Primary Care Nurse, GI Nurse, Scheduler, etc.)?

2. Is fecal occult blood testing (FOBT) or the fecal immunochemical test or “FIT” for cancer screening used at your facility? (NO REAL DETAILS NEEDED BEYOND FIT, FOBT, or BOTH)
3. Tell me how your facility handles FIT or FOBT positive results?
   a. Probe: Who is involved in this process and what are their roles?
   b. Probe: Are patients with FIT or FOBT referred for a gastroenterology clinic appointment or referred directly to colonoscopy?
4. Are you familiar with the diagnostic colonoscopy reminder for patients with FIT or FOBT positive results?
5. What, if anything, has been helpful in addressing these reminders for FIT or FOBT positive patients?
   a. Probe: What have you found helpful in getting FIT or FOBT positive patients colonoscopies?
   b. Probe: What changes has your facility has made to follow up positive FIT or FOBT results since these reminders became available?
   c. Probe: Who______ (this probe is intended to understand who at a given facility handles these results, the blank allows the interviewer flexibility in phrasing).
6. Please describe any barriers that you perceive exist that prevent FIT or FOBT positive Veterans at your facility from getting timely colonoscopy?

   Probe: How do you know when a colonoscopy has been completed?

   Probe: if a patient cancels/no show, will the colonoscopy get rescheduled?

   Probe: if patient refuses, what are some of the reasons?
7. What happens when you send FIT OR FOBT positive patients to colonoscopies in the community?

   If yes:
   a. What have you found to be helpful in getting patients colonoscopies in the community?
   b. What have you found difficult in getting patients colonoscopies in the community?
   c. How do you get the information from the colonoscopy back from community providers?
8. Do you have any suggestions to improve the rate of colonoscopy after FIT OR FOBT?
9. Besides arranging colonoscopies, are there other difficulties in getting the diagnostic colonoscopy reminders resolved?

Thank you for participating in this interview.
